# Resting-State Functional Connectivity Signatures of Apathy in Community-Living Older Adults

**DOI:** 10.3389/fnagi.2021.691710

**Published:** 2021-06-25

**Authors:** Jung Yun Jang, S. Duke Han, Belinda Yew, Anna E. Blanken, Shubir Dutt, Yanrong Li, Jean K. Ho, Aimée Gaubert, Daniel A. Nation

**Affiliations:** ^1^Institute for Memory Impairments and Neurological Disorders, University of California, Irvine, Irvine, CA, United States; ^2^Department of Family Medicine, University of Southern California, Los Angeles, CA, United States; ^3^Department of Psychology, University of Southern California, Los Angeles, CA, United States; ^4^Department of Neurology, University of Southern California, Los Angeles, CA, United States; ^5^School of Gerontology, University of Southern California, Los Angeles, CA, United States; ^6^Department of Psychological Science, University of California, Irvine, Irvine, CA, United States

**Keywords:** apathy, older adults, functional connectivity, resting-state fMRI, reward processing, salience processing

## Abstract

Apathy predicts poor outcomes in older adults, and its underlying neural mechanism needs further investigation. We examined the association between symptoms of apathy and functional connectivity (FC) in older adults without stroke or dementia. Participants included 48 individuals (mean age = 70.90) living independently in the community, who underwent resting-state fMRI and completed the Apathy Evaluation Scale (AES). Seed-to-voxel analysis (cluster-level p-FDR <0.05, voxel threshold *p* < 0.001) tested the association between AES scores and the whole-brain FC of brain regions involved in reward- and salience-related processing. We found that AES scores were negatively associated with FC of the right insula cortex and right anterior temporal regions (124 voxels, *t* = −5.10) and FC of the left orbitofrontal cortex and anterior cingulate regions (160 voxels, *t* = −5.45), and were positively associated with FC of the left orbitofrontal cortex and left lateral prefrontal (282 voxels, *t* = 4.99) and anterior prefrontal (123 voxels, *t* = 4.52) regions. These findings suggest that apathy in older adults may reflect disruptions in neural connectivity involved in reward- and salience-related processing.

## Introduction

Apathy is a multi-domain syndrome, with motivational deficits and declines in goal-directed behavior as its chief symptoms (Marin, 1991). Research has shown that apathy increases with age (Brodaty et al., 2010) and is associated with poor outcomes in older adults, such as difficulties in daily functioning and decreased quality of life (Tierney et al., 2018). Growing evidence indicates a link between apathy and incident dementia in healthy older adults (van Dalen et al., 2018; Bock et al., 2020) and provides support for the hypothesis that apathy may be a prodromal feature of Alzheimer's disease spectrum (Munro et al., 2015). These findings highlight the need to investigate the association between apathy and brain changes that have unfavorable implications for healthy aging.

Neuroimaging studies of apathy have predominantly focused on individuals with brain diseases or psychiatric conditions, including stroke (Kang and Kim, 2008; Onoda et al., 2011; Rochat et al., 2013; Kumral et al., 2019), neurodegeneration (Apostolova et al., 2007; Marshall et al., 2007; Reijnders et al., 2010; Tunnard et al., 2011; Eslinger et al., 2012; Carriere et al., 2014; Jones et al., 2019), and late-life depression (Lavretsky et al., 2007; Alexopoulos et al., 2013; Yuen et al., 2014). Reviews integrating structural and functional correlates of apathy across different disorders have identified regions commonly associated with apathy, including the anterior cingulate cortex (ACC), ventral striatum (nucleus accumbens; NAcc), thalamus, orbitofrontal cortex (OFC), and insula (IC) (Kos et al., 2016; Le Heron et al., 2018; Pimontel et al., 2020). These are considered key nodes in networks responsible for decision-making, initiating effortful activities, and reward- and salience-related processing (Kos et al., 2016; Le Heron et al., 2018; Pimontel et al., 2020).

In healthy older adults, one study investigated the association between apathy and executive function, and its relation to frontal-striatal connectivity (Kawagoe et al., 2017). Authors found that apathy is negatively associated with executive function, and that older adults with apathy and low executive function showed decreased connectivity in the frontal pole and NAcc (Kawagoe et al., 2017).

Researchers have conceptualized apathy in terms of “effort-based decision making for rewards” for a goal or activity (Husain and Roiser, 2018). In accordance with this approach, we hypothesized that apathy in older adults would be associated with alterations in functional connectivity (FC) of these regions underlying decision-making, effort, and reward and salience processing. For instance, disrupted FC may represent failures in recognizing a given activity as rewarding and in allocating effort to initiate the activity (Pimontel et al., 2020). These failures may be described in behavioral terms as a lack of motivation, which is the hallmark of apathy. The current study examined the association between apathy and whole-brain FC measured by resting-state functional MRI (rs-fMRI) in community-dwelling, functionally independent older adults. We explored brain structures identified by the recent comprehensive reviews (described above) as seed regions of interest (ROIs), as evidence from disorders prevalent in older adults could be informative in studying healthy older individuals.

## Materials and Methods

### Participants

Participants included 48 older adults [age 59–90; mean age = 70.90 (*SD* = 7.88); 62.5% male] from the Vascular Senescence and Cognition (VaSC) Study, living in the greater Los Angeles area. Exclusion criteria for the VaSC Study included current depression or history of major psychiatric disorder, clinical stroke, diagnosis of dementia by history or during in-person exam [Dementia Rating Scale-2 (DRS) total score < 126], traumatic brain injury with loss of consciousness (15 minutes or longer), substance abuse and/or dependence, and systemic conditions (e.g., metastatic cancer) or medications (e.g., narcotic analgesics) that may affect cognition.

### Procedures

Informed consent was obtained from all participants. The Institutional Review Board at University of Southern California approved the VaSC study protocol. Eligibility screening was conducted by phone. Participants completed a neurocognitive assessment, neuropsychiatric questionnaires, and MRI scans. Informants reported on participants' daily functioning, which was used to aid dementia screening. Participants received monetary compensation for study participation.

### Measures

Apathy symptoms were measured using the Apathy Evaluation Scale (AES). AES is an 18-item questionnaire, with scores ranging 18–72. Higher scores indicate greater symptoms of apathy. Depressive symptoms were assessed by the 30-item Geriatric Depression Scale (GDS). Global cognitive functioning was measured by the DRS total score.

Participants eligible for MRI underwent structural and functional MRI, using the 3T Siemens Prisma scanner with 20-channel head coil. For structural data, high-resolution T1-weighted images were acquired, using 3-dimensional magnetization-prepared rapid gradient-echo (MPRAGE) sequences. Rs-fMRI scans comprised 140 contiguous echo-planar imaging (EPI) functional volumes (TR = 3,000 ms, TE = 30 ms, FA = 80°, 3.3 × 3.3 × 3.3 mm voxels, matrix = 64 × 64, FoV = 212 mm, 48 slices). Participants were asked to lie still with their eyes open.

### Analyses

All images were preprocessed and analyzed using the CONN Toolbox (Whitfield-Gabrieli and Nieto-Castanon, 2012; http://www.nitrc.org/projects/conn/).

#### fMRI Data Preprocessing

The CONN default preprocessing steps included realignment to first scan, correcting for slice-timing discrepancies, spatial normalization to the 2 mm isotropic Montreal Neurological Institute (MNI) template, and spatial smoothing with an 8 mm full-width half-maximum (FWHM) Gaussian kernel. Further removal of confounding signals was conducted through nuisance regression. Regressors included (1) 6 motion parameters (3 rotation and 3 translation) estimated from the realignment step and their first-order derivatives, (2) principal components from the white matter and the cerebrospinal fluid masks, following an anatomical component-based noise correction strategy (CompCor), and (3) outlier scans identified using a threshold of framewise displacement > 0.5 mm and global intensity z-score of 3 to effectively scrub motion artifacts. Finally, a 0.008–0.09 Hz band-pass filter and linear detrending were applied.

#### Seed-to-Voxel Analyses

CONN provides anatomically-derived ROIs based on the Harvard-Oxford brain atlas. Mean activity in the ROIs were computed by averaging blood-oxygen level dependent (BOLD) time series from all voxels within each ROI and used as the reference. ROIs included the ACC, NAcc, OFC, thalamus, and the IC. ROIs were examined bilaterally, except for the ACC. FC maps were created for each participant based on Fisher's r-to-z transformed correlations between the mean time series in each ROI and the time series of every voxel in the whole brain. ROI FC maps were then regressed on AES scores in the general linear model, adjusting for age. Voxel-level threshold was *p* < 0.001. To correct for multiple comparison, cluster threshold of p-FDR < 0.05 was applied. Two-tailed tests were used to determine statistical significance.

## Results

On average, participants were well-educated (mean education = 16.02 years, *SD* = 2.19), endorsed minimal levels of depressive symptoms (mean GDS = 3.65, *SD* = 4.09), and showed no cognitive impairment on testing (mean DRS total = 140.33, *SD* = 3.19). Mean AES score was 24.93 (*SD* = 4.82).

### Apathy Is Related to the Functional Connectivity of the Insula and Orbitofrontal Cortices

**Figure 1:**
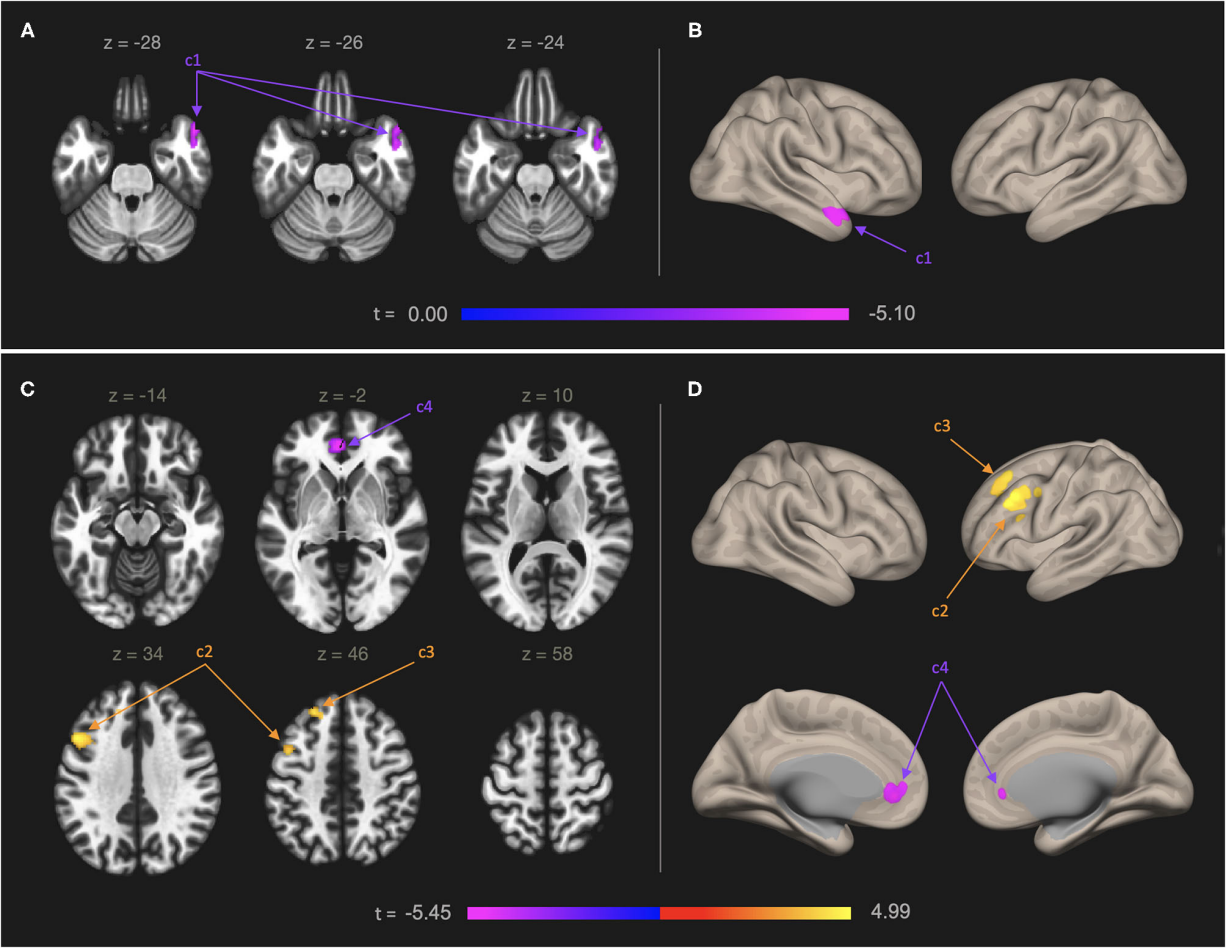
Functional connectivity map of the right IC **(A,B)** and left OFC **(C,D)** in relation to self-reported apathy symptoms. Models were adjusted for age. Left panel: Results shown in axial slices of the brain. Right panel: A 3-dimentional rendering of the inflated brain showing the results. Numbers on the color bars indicate peak voxel t-statistics. Statistical significance was determined by cluster p-FDR < 0.05 and voxel-level threshold *p* < 0.001. **(A,B)** Self-reported symptoms of apathy were negatively associated with neural connectivity between right IC ROI and a cluster of 124 voxels in the left anterior temporal regions (cluster 1, “c1”), including the right temporal pole (84 voxels) and right anterior middle temporal gyrus (25 voxels). **(C,D)** Self-rated symptoms of apathy were positively associated with neural connectivity between the left OFC ROI and a cluster of 282 voxels in the left lateral prefrontal regions (cluster 2, “c2”), including left middle frontal gyrus (268 voxels) and a cluster of 123 voxels in the anterior prefrontal regions (cluster 3, “c3”), including left superior frontal gyrus (73 voxels) and the left frontal pole (34 voxels). The symptoms were negatively associated with neural connectivity between the left OFC and a cluster of 160 voxels in the anterior cingulate regions (cluster 4, “c4”), including the left paracingulate gyrus (83 voxels) and the ACC (57 voxels).

Table 1 and Figure 1 present results from seed-to-voxel analysis with the right IC and left OFC ROIs as the seeds. AES scores were negatively associated with FC of the right IC and a cluster in the anterior temporal regions [cluster 1: 124 voxels; peak voxel MNI coordinates: 52, 14, −28; *t*_(45)_ = −5.10], including the right temporal pole (84 voxels) and right anterior middle temporal gyrus (25 voxels). For the left OFC ROI, AES scores were positively associated with FC in the left lateral prefrontal regions [cluster 2: 282 voxels; peak voxel: −48, 16, 34; *t*_(45)_ = 4.99], including left middle frontal gyrus (268 voxels), and the anterior prefrontal regions [cluster 3: 123 voxels; peak voxel: −22, 36, 46; *t*_(45)_ = 4.52], including the left superior frontal gyrus (73 voxels) and the left frontal pole (34 voxels). AES scores were negatively associated with FC of the left OFC and the anterior cingulate regions [cluster 4: 160 voxels; peak voxel: −2, 44, −2; *t*_(45)_ = −5.45] including the left paracingulate gyrus (83 voxels) and the ACC (57 voxels).

**Table 1:** Results from seed-to-voxel analysis of apathy symptoms and functional connectivity of the right insula and left orbitofrontal cortex.

**Seed ROI**	**Cluster**	**Location (MNI)**			**Cluster size**	**Brain regions**	**Number of voxels**	***t***
		**x**	**y**	**z**				
Right insula	1	52	14	−28	124	R temporal pole	84	−5.10
						R anterior middle temporal gyrus	25	
						R anterior superior temporal gyrus	3	
Left orbitofrontal cortex	2	−48	16	34	282	L middle frontal gyrus	268	4.99
						L precentral gyrus	9	
						L pars opercularis	4	
	3	−22	36	46	123	L superior frontal gyrus	73	4.52
						L frontal pole	34	
						L middle frontal gyrus	4	
	4	−2	44	−2	160	L paracingulate gyrus	83	−5.45
						Anterior cingulate	57	
						R paracingulate gyrus	6	

*Voxel-level p < 0.001, cluster p-FDR < 0.05*.

We did not find any significant clusters of voxels for the ACC, right OFC, left IC, bilateral NAcc, and bilateral thalamus ROIs.

## Discussion

The current study investigated the association between apathy and resting-state FC of the ROIs derived from the neural correlates of apathy in the context of neurological and psychiatric disorders. To our knowledge, this is one of the first studies to evaluate apathy symptoms in community-dwelling, independently functioning older adults who are free of clinical stroke, dementia, or ongoing depression. We found that elevations of apathy-related symptoms were associated with reduced FC between the IC and anterior temporal regions and between the OFC and the anterior cingulate regions, and with increased FC between the OFC and regions within the dorsolateral prefrontal cortex (DLPFC). We did not find any relationship between apathy and FC of the other ROIs, including the NAcc, and thalamus.

The OFC and ACC are major cortical structures in the reward network, involved in processing higher-level motivators and facilitating goal-directed behaviors toward the optimal outcome (Liu et al., 2011; Pimontel et al., 2020). The OFC is responsible for integrating the incoming information to appraise and predict rewards and losses (Pimontel et al., 2020), whereas the ACC plays an important role in decision-making based on cost-benefit analysis, prior to exerting effort (Shenhav et al., 2013; Le Heron et al., 2018). Deficits in connectivity between these regions may result in attenuated reward sensitivity, a possible underlying mechanism of apathy (Tay et al., 2020). Alternatively, the OFC is involved in modulating motivational salience (Rothkirch et al., 2012), and the ACC is one of the primary regions in the salience network. Changes in OFC-ACC connectivity may represent dysfunctions in salience processing.

The IC is a hub of the salience network, which may provide motivational context for goal-directed behavior by identifying the most relevant stimuli among massive internal and environmental inputs competing for attention (Yuen et al., 2014; Pimontel et al., 2020). Given its proximity and connectivity to the amygdala and the OFC, anterior temporal regions are thought to support socio-emotional functioning, and temporal pole damage has been implicated in socio-emotional impairment, including decreased interest in others and loss of empathy (Olson et al., 2007). The decreased connectivity of the IC and the anterior temporal regions might suggest inadequate processing of socio-emotional stimuli as salient, which could subsequently lead to a failure to recruit and engage other networks to generate behavior.

Finally, the DLPFC is thought to be part of the networks involved in attentional control processes, initiating and monitoring behavior (Tay et al., 2020). The association between apathy symptoms and increased connectivity between the OFC and the DLPFC regions might reflect FC alterations that are compensatory, possibly a greater reliance on attentional resources in response to deficits in reward-related processing.

Our conceptual framework allowed us to consider these findings in the context of specific functional processes and networks. However, it is entirely possible that the altered connectivity between regions we found may be part of neural networks not discussed here. Given the relatively mild apathy symptoms noted in our sample, lack of subcortical findings might imply that regions such as the NAcc and the thalamus are involved in more severe symptoms (e.g., apathy observed in neurological or psychiatric disorders). Other study limitations include the cross-sectional design and reliance on a well-educated and healthy sample, which may limit generalizability.

Despite limitations, the current findings underscore the need for assessment of socio-emotional functioning in older adults as a standard practice, as the declines may be linked to changes in brain function. Findings may also inform future research to test the utility of network connectivity disruptions in evaluating risk for neurobehavioral symptoms in older adults. Apathy is a common neurobehavioral problem in mild cognitive impairment (Ma, 2020) and mild behavioral impairment (Mortby et al., 2017). Future studies may learn from exploring FC changes related to apathy in these prodromal stages of dementia. Finally, the current findings may contribute to development of intervention strategies targeting processes underlying apathy in older adults.

## Data Availability Statement

The raw data supporting the conclusions of this article will be made available by the authors, without undue reservation.

## Ethics Statement

The studies involving human participants were reviewed and approved by Institutional Review Board, University of Southern California. The patients/participants provided their written informed consent to participate in this study.

## Author Contributions

JJ designed the study, conducted data analysis, interpreted the results, and prepared the manuscript. SH, BY, AB, SD, YL, JH, and AG conducted data analysis, interpreted the results, and contributed to manuscript preparation. DN designed the study, conducted data analysis, interpreted the results, prepared the manuscript, and supervised this research. All authors contributed to the article and approved the submitted version.

## Conflict of Interest

The authors declare that the research was conducted in the absence of any commercial or financial relationships that could be construed as a potential conflict of interest.
